# Therapists’ and Patients’ Perspectives on Therapeutic Dynamics Leading to Therapy Failure in Forensic Addiction Treatment

**DOI:** 10.3389/fpsyt.2019.00879

**Published:** 2019-12-17

**Authors:** Jan Querengässer, Lena Langenstück, Klaus Hoffmann

**Affiliations:** ^1^Department of Psychology, University of Konstanz, Konstanz, Germany; ^2^LWL-Academy for Forensic Psychiatry, Herne, Germany

**Keywords:** forensic psychiatry, substance abuse, therapy failure, offender treatment, treatment dynamics, therapeutic process, addiction treatment

## Abstract

**Background:** Among drug- or alcohol-addicted offenders under forensic treatment, therapy failure is a potent predictor of substance-related re-delinquency. Given this evidence, high drop-out rates pose a major problem in forensic addiction treatment in Germany. Legal preconditions for a premature discharge due to therapy failure are defined, and behavioral correlates are well described, but the precedent dynamics between patients and therapists have rarely been analyzed. The present study intended to shed light upon the subjective perception of the treatment course prior to therapy failure.

**Methods:** Applying parallel questionnaires and structured interviews, patients’ and therapists’ perspectives on perceived reasons for therapy failure were retrospectively investigated and compared to each other on a dyadic level. Following this predominantly qualitative and explorative approach, the examination of 32 dyads could be realized; 13 patients with regular (i.e., successful) therapy termination served as controls. All patients had been treated within two specialized forensic addiction hospitals in the German federal state of Baden-Württemberg and were assessed shortly before discharge took place.

**Results:** As expected, patients’ and therapists’ perspectives differed largely on perceived reasons for failure. In most cases, they appeared to have very different views on what happened during treatment and why therapy eventually failed. Patients mentioned psychological tension and aggressiveness, frequent quarrels with fellow patients, and a bad therapeutic environment as most important reasons for therapy failure. Therapists highlighted patients’ unwillingness to make an effort or to change behavior. The analysis of patients’ narratives regarding how to explain the negative treatment course confirmed pre-assumptions on predominantly negative feelings and attitudes towards the clinic. The precedent dynamics of therapy failure were shown to be highly individual. However, despite varying notably, a cluster analysis revealed that they seemed to follow “typical patterns” that could partially be linked to patients’ characteristics.

**Conclusions:** A better understanding of treatment dynamics during forensic addiction therapy is a prerequisite for the avoidance of therapy failure with negative effects on re-delinquency. It seems that the incapacity to establish a common frame of reference for assessing the therapy process could be one of the major reasons why treatment dynamics take on a life of their own towards a disruption of the therapeutic relationship, leading to therapy failure. The knowledge of “typical” risk patterns towards therapy failure could facilitate early therapeutic measures.

## Introduction

Within the German legal framework, courts shall make a custodial addiction treatment order (sec. 64 of the German Criminal Code—*StGB*) if an unlawful act is committed by an alcohol- or drug-addicted offender. In 2017, courts applied this rule, which is unique to Germany, on 2,829 individuals. The comparison to 33,285 offenders that have been convicted to serve a prison sentence (without probation) results in a ratio of 1 to 12 (1). However, as only offenses above a certain threshold of severity justify a treatment order according to sec. 64 *StGB*, a more adequate reference is the number of prison sentences with a duration of more than 2 years (n = 9,450), resulting in a ratio of 1 to 3 (1). This figure underlines the importance of forensic addiction treatment orders within the German criminal justice system.

The precondition for applying an addiction treatment order is an unlawful act that must be attributed to the offender’s substance addiction, be it directly (e.g., violent acts during intoxication) or indirectly (e.g., robbery to finance the purchase of drugs, drug dealing itself). In these cases, the offenders are sent to specialized hospitals where addiction treatment takes place. Forensic addiction hospitals are structurally and locally separated both from the regular mental health system and from the regular prison system. Instead, inmates are treated in a milieu therapy approach with higher degrees of freedom but with higher requirements for change motivation as well. In contrast to “common” addiction treatment, which is financed by the public health insurance system, forensic facilities are funded directly by the government and dispose of a higher security level. With regards to content, forensic addiction therapy focuses on the complex relationship between delinquency and addictive behavior.

While the figures of patients under a forensic addiction treatment order have been growing for many years in Germany (1), the proportion of premature therapy termination due to a marginal prospect of success (acc. to sec. 67d V *StGB*) remains stable by approximately 50% (2). In general, there is little research interest in failure of psychotherapy (3) that is no surprise: A premature termination implies a frustrating experience both for patients and therapists, along with negative emotions and aggression in general and the feeling of failure in particular (4, 5). The vast majority of forensic addiction patients with premature therapy termination are rereferred to the prison system, as they must serve a concurrent prison sentence. Therefore, therapy failure in this context implies more severe consequences than in other therapeutic contexts. Empirical evidence shows that therapy failure is a potent predictor of substance-related re-delinquency [(6–8), as a meta-analysis on forensic therapy in general: (9)]. Hence, reducing the proportion of premature therapy terminations is of major interest.

To address this interest, scientific endeavors have focused on the reliability of treatment prognoses for many years. An understandable perspective, as one of the preconditions to apply a forensic addiction treatment order, is a positive treatment prognosis, and courts are obliged to base the decision exclusively on the offender’s behavior and personal background. However, empirical research did not meet the expectations, as only few and weak person-related predictors as younger age, previous delinquency, the type and severity of the index offense, occupational status prior to conviction, absence of educational qualification and comorbidity (especially psychosis and personality disorder) could be identified [(10–12), as a summary of previous studies: (13)].

From a therapeutic point of view, this retrospective and person-centered perspective is not exhaustive. A growing body of evidence indicates that context and setting factors show moderate to strong effect sizes concerning the effectiveness of psychotherapy [(14), for the German forensic system: (12)]. These effect sizes are notably higher than those of technical or professional factors. Hence, the treatment dynamics between patients and therapists that precede a premature therapy termination should be analyzed in more detail.

Within the forensic system, there is some descriptive knowledge of the reasons why forensic hospitals demand premature therapy termination (which is a final decision taken by the supervising court): substance use, escapes or other forms of a severe breach of rules (5,, 16). However, these “manifest” phenomena could better be characterized as occasions, as they do not explain the underlying causes of premature discharge. Moreover, it seems as if forensic hospitals use such observable behaviors as a welcome support of their line of argument.

Every premature therapy termination should be seen as the endpoint of a dysfunctional treatment course and not as a single event. Following this presumption, we conducted a pilot study and analyzed 39 letters in which the forensic hospital demands premature therapy terminations (see above). A cluster analysis of the described causes and occasions revealed three “typical patterns” of treatment dynamics preceding a premature therapy termination (17): the first pattern was characterized by the patients’ passive refusal, the second by confrontation and acting out and the third by impulsive refusal.

However, even that study, which was based solely on the analysis of existing documents, followed the “objective” therapeutic view, as demonstrated *via* correspondence with the court. Patients’ and therapists’ subjective perceptions of the reasons for therapy failure in forensic addiction treatment and preceding therapy dynamics have not yet been investigated.

As a measure of internal quality assurance, we conducted a study that intended to shed light upon the subjective perception of the treatment course prior to therapy failure and other related areas as therapy goal attainment and learning experiences. By applying a set of semistructured interviews and questionnaires, several topics were investigated: evaluation of reasons for premature termination, attitudes towards and conformity to therapy requirements, (self-)criticism, therapy goal attainment and learning experiences. Many of the study’s findings have been published in detail in journals published in the German language (18–23), while others remained unpublished.

The present article presents the study’s findings concerning the central question: How do patients and their therapists with premature therapy termination explain the precedent dynamics subjectively?

To draw a comprehensive picture of our results, in the present article, we focus on the formerly unpublished analyses but will first give an overview over some of the materials previously published in the German language (21, 23) to make them accessible to non-German readers for the first time. The additional and original information of the present article is a content analysis of narratives, the way in which patients subjectively explain the reasons, and a cluster analysis based on the quantitative data on reasons for premature therapy termination. Due to the absence of existing literature on the topic, we abstained from distinct hypotheses and exploratorily examined the research assumption that patients and therapists differ in the subjective evaluation of preceding treatment dynamics. Nonetheless, we also intended to replicate the cluster results derived from our pilot study (17).

## Methods

The study was designed as a multicenter cross-sectional retrospective study combining semistructured interviews and questionnaires. Two levels of comparison were intended: 1) within-subject: patients and their therapists were interrogated concerning their perspective on the treatment course; and 2) between-subject: patients with regular and premature therapy termination were compared.

In two public forensic clinics run by the German federal state of Baden-Württemberg, a convenience sample of patients (criterion: therapy termination within the 1.5-year period of investigation) was recruited. Sixty-eight patients were asked to participate, and 50 gave informed consent and were included. The 37 included patients with premature therapy termination formed group A. For 34 patients outside of that group, information from the respective therapists could be obtained (patients had previously given informed consent and released therapists from medical confidentiality). For organizational reasons, the control group B of 13 patients with regular (i.e., successful) therapy termination was derived from only one clinic.

All patients were diagnosed with substance addiction (acc. to chapter F1 of the ICD-10): 17 due to alcohol, 16 due to opioids, 4 due to cocaine, and 13 due to polytoxic addiction problems. A total of 18 had been convicted of drug dealing, 11 for committing physical assault, 10 for engaging in other violent acts (e.g., robbery), 5 for engaging in theft, 3 for committing (attempted) homicide, 2 for committing sexual offences, and 1 other. Between groups A and B, no significant differences concerning diagnoses or offences could be found. Group A patients were older than group B patients [mean age of 37.4 years ( ± 9.66 SD) vs. 32.0 years ( ± 5.43 SD)], whereas group B showed a longer treatment course than group A [30 months ( ± 5.53 SD) vs. 14 months ( ± 9.47 SD)].

Data collection was performed *via* a combination of a self-developed questionnaire covering quantitative information on perceived causes and occasions for premature therapy termination and attitudes towards and assessed conformity to therapy requirements. The questionnaire covered 28 statements concerning motives and possible causes of treatment termination (e.g. *“I often quarreled with my fellow patients”*) and 8 possible occasions for premature therapy termination (e.g. *“Substance use on the ward”*), both basing on previous literature (5, 15–17). Subsequently, a semistructured interview was administered covering an assessment of possible causes and occasions for premature therapy termination, subjective narratives, (self-)criticism, therapy goal attainment, and learning experiences.

Patients were interrogated by a researcher who had not been involved with patients’ therapy. The interviews each lasted 30 to 60 min, including the pen-and-paper application of the questionnaire, and took place in a confidential and separate room within the ward. Patients obtained a reward of 5 Euro. For group A patients, a questionnaire parallel to the patient form of the data collection material was given to the respective therapist.

As the study was primarily performed within the context of an internal quality assurance evaluation, no ethical approvals were obtained. The study did not include any aspects of interventions, and the head offices of the involved hospitals were informed in detail and declared their approval. Informed written consent was requested from all participants, including a detailed description of the interrogation procedures and the secondary research purpose of the interrogation. Sociodemographic data were collected using basic data from court and medical files.

The collected data were revised, coded, tabulated, and entered into a PC *via* Microsoft Excel and the Statistical Package for the Social Sciences (SPSS 20). Data were presented, and suitable nonparametric analysis was performed according to the type of data obtained for each parameter:

- Descriptive statistics:
  1. Mean and standard deviation ( ± SD) for numerical data.
  2. Frequency and percentage for nonnumerical data.
- Analytical statistics:
  1. The Mann-Whitney U-test was used to assess the statistical significance of the difference between study group means.
  2. Chi-square tests and Fisher’s exact tests were used to examine the relationship between two qualitative variables. As an effect size, we calculated ϕ.
  3. Correlation analysis (using Spearman’s ρ): To assess the strength of association between two quantitative variables.
  4. A hierarchical cluster analysis was calculated using a transformed ϕ-4-point-correlation as a measure of distance. A complete-linkage procedure on the grouping of patients was used and a three-cluster solution was chosen for further analysis using a divisive strategy.

## Results

### Overview of the Results Previously Published in the German Language

#### Possible Causes for Premature Therapy Termination

The first topic of the study addressed a set of 28 statements concerning motives and possible causes of treatment termination (see **Table 1**, upper section). These statements were rated by patients and their therapists in two ways (n = 29 dyadic ratings could be realized). First, they indicated on a visual analogue scale the extent of their agreement on each statement. Second, they specified which of the statements includes a reason for their premature therapy termination.

**Table 1:** Statements and occasions indicating possible reasons for premature therapy termination; proportion of patients rating the statement as relevant; cluster differences (construct-related).

Statement or occasion^2^		Cluster group^1^			Test statistics (each df = 2)	
	Total	Substance related	Motivational deficiency	Interactional		
	n = 37	n = 7	n = 10	n = 14	Chi²	p^3^
I am satisfied with therapy in general (inv.)	20%	29%	30%	14%	1.1	.60
My family made it too easy for me during therapy	3%	–	11%	–	2.4	.30
My friends and acquaintances supported me during therapy (inv.)	6%	–	22%	–	5.0	.08°
During therapy, I dealt intensively with my offence (inv.)	20%	–	50%	14%	6.9	.03*
I was not keen on making an effort during therapy	14%	14%	30%	7%	2.3	.32
In my case, I do not believe in therapy success	15%	–	50%	–	12.0	.002**
My fellow patients did affect me negatively	26%	43%	–	43%	6.0	.05*
My family supported me during therapy (inv.)	–	–	–	–	–	
I was ready to change important areas of my life (inv.)	6%	14%	10%	–	1.9	.39
Regular therapy termination is of no use for me	6%	-	20%	–	4.5	.11
I often felt aggressive or stressed	43%	14%	40%	64%	4.9	.09°
I was adequately informed about the possible duration of therapy (inv.)	23%	14%	50%	14%	4.5	.11
I think the prison would have been a better place for me	9%	–	20%	7%	2.1	.36
I felt overstrained by therapy	14%	14%	30%	7%	2.3	.32
I have other somatic difficulties to deal with, which impaired me during therapy	20%	–	40%	21%	3.8	.15
My fellow patients supported me during therapy (inv.)	6%	14%	–	7%	1.4	.49
I often quarreled with my fellow patients	26%	14%	–	50%	8.2	.02*
Therapists displayed enough patience with me (inv.)	12%	–	10%	23%	2.2	.33
I always got along with my therapist(s) (inv.)	21%	–	10%	43%	6.2	.04*
I don’t feel fine on the ward	40%	14%	40%	57%	3.5	.17
My motivation to finish therapy regularly was very labile	27%	14%	40%	29%	1.3	.52
From time to time, I simply didn’t attend therapy sessions	6%	14%	10%	–	1.9	.39
There has never been a gap between what I said and what I did (inv.)	14%	–	–	36%	7.2	.03*
From time to time, I took some liberties with the things I reported to my therapists	11%	14%	10%	14%	.1	.95
I feel very connected to friends that regularly consume drugs or commit offences	17%	57%	10%	7%	8.3	.02*
I actually never wanted to start therapy	9%	–	30%	–	7.0	.03*
If I had more time for therapy, I would have finished it regularly	12%	14%	11%	14%	.1	.97
It is my own will to skip therapy	24%	–	70%	8%	14.5	.001**
Substance use in general	30%	100%	10%	21%	16.8	>.001**
Substance use on the ward	11%	57%	–	–	15.8	>.001**
Importing substances to the ward	8%	43%	–	–	11.4	.003**
Sharing substances with fellow patients	3%	14%	–	–	3.5	.17
Substance dealing on the ward	3%	14%	–	–	3.5	.17
Escape(s)	19%	14%	40%	14%	2.6	.28
Offences committed during escape	3%	–	10%	–	2.2	.34
Severe breach of rules	19%	43%	10%	21%	2.6	.28

^1^n = 6 missing values due to exclusively 0-values or n > 3 single missing values.

^2^inv.: statement was worded inversely.

^3^° if p < .1, * if p < .05, ** if p < .01.

With respect to the statements being subjectively viewed as appropriate reasons for therapy termination, the patients indicated more reasons overall for treatment termination being appropriate than the therapists did. First and foremost, the patients mentioned their own psychological tension and aggressiveness (43% out of all patients), frequent quarrels with fellow patients (26%), and various other negative influences in relation to a bad therapeutic environment as directly relevant to their therapeutic failure (21). In contrast, the therapists highlighted the patients’ own behaviors as the principal cause of failure (e.g., a lack of willingness to change: 37%). On the within-group level, patients’ and therapists’ ratings significantly differed in eight out of the 28 statements (Fisher’s exact test statistics with p < .05). On the dyadic level, concordant entries were rare: a significant concordance was found only on three out of 28 statements, with.41* ≤ ϕ ≤ .61** (21).

Finally, index patients’ ratings on the 28 statements were compared to those of another group of patients with regular (i.e., successful) therapy termination (group B as described above). This between-group comparison revealed significant differences in 10 out of 28 applicable statements (Mann-Whitney U test statistics with p < .05). Interestingly, all statements concerning the therapeutic relationship were rated significantly higher by patients with regular therapy termination (23).

#### Occasions for Premature Therapy Termination

The second topic focused on possible occasions for premature therapy termination, covering several forms of drug consumption, drug dealing on the ward and escapes (see **Table 1**, lower section). Patients and therapists then rated a) if the mentioned occasions did occur and b) if they were relevant as a reason for the premature termination of therapy. As expected, on the within-group comparison level, the proportion of patients’ and therapists’ entries did not differ (Fisher’s exact test statistics each n.s.), and concordance was high, with .49* ≤ ϕ ≤ 1.00**.

Surprisingly, even on these rather “objective” facts, concordance was low concerning the subjective rating if the occasion was seen as a relevant reason for premature therapy termination. Only concerning escapes, ratings revealed significant concordance between patients and therapists: ϕ = .91* (20).

Another unexpected finding was the absence of significant differences between the index group and patients with regular therapy termination (Fisher’s exact test statistics each n.s.). Contrary to expectations, some of the occasions descriptively occurred *more often* among successful patients (e.g., 69% reported substance abuse during treatment, while only 54% of the index group did so).

### Original and Previously Unpublished Analyses and Material

#### Content Analysis of Narratives

At the beginning of the semistructured interview, patients were asked to briefly summarize the reasons for therapy termination in their own words. Disappointment, the feeling of being treated in an unfair manner or a lack of feeling understood played a role in 14 out of the 37 analyzed narratives (38%). More drastic words (*“deviled”, “they were shittin’ me”, “deceived”*) were used by 10 patients (27%), and a loss of confidence was mentioned by four patients (11%).

Fourteen patients (38%) mentioned substance abuse during treatment in their narratives. Interestingly, half of them attributed the reasons for substance abuse externally (e.g., *“I relapsed because I couldn’t see an end after about three years of treatment.”*). Nine patients (24%) addressed escapes, and again, most of them (n = 5) used external attributions as an explanation (e.g., *“They bullied me due to a tic. Instead of talking to my therapist, I escaped.”*).

As described above, the second topic of the questionnaire dealt with occasions for premature therapy termination in a structured way. Between that chapter and the narratives, there was 100% conformity with respect to escapes: all nine patients indicated escapes in both ways.

Concerning substance abuse, conformity was lower: out of the 20 patients indicating substance use in the structured part, only 14 (70%) mentioned substance use in their narratives. Hence, for six patients (30% of all who relapsed), substance use during treatment did not play a role in their subjective concept.

#### Cluster Analysis of Reasons for Premature Therapy Termination as Mentioned by Patients

Based on patients’ ratings, if one of the statements concerning possible causes and occasions (see above) for premature therapy termination was subjectively relevant as a reason for their own premature therapy termination, a cluster analysis was performed, and group A patients were clustered, resulting in a three-group cluster solution. The three groups differed significantly in 12 of the 36 possible reasons (see **Table 1**) with *p* < .05 (on two other statements, differences were found with *p* < .1). These differences were used to describe and name the three groups. To understand the groups correctly, it is necessary to bear in mind that they were not formed based on the occurrence of the occasions or the degree of agreement with the statements but on the rating, if the possible reasons played a role in the subjective pattern of explanation concerning premature therapy termination. Therefore, the clusters form prototypical patterns of explanations instead of patterns of behaviors.

Group I (n = 7): substance-related pattern of explanation. In this group, all patients mentioned the consumption of psychoactive substances on at least one occasion during treatment—most of them on the ward, and in almost one out of two cases, patients imported the substances on their own. Solidarity with the drug milieu is mentioned by the majority, whereas other topics related to the mere therapeutic process were not mentioned once. Compared to the other groups, this group had the fewest number of cases in which aggressiveness and tension were mentioned.

Group II (n = 10): motivational deficiency pattern of explanation. The second group is characterized by the desire to drop therapy. Only one of the group members mentioned substance consumption, none of them reported struggles with fellow patients or negative influences from them. Instead, the group showed the highest percentages regarding therapy-related topics and (a lack of) assistance from friends and acquaintances.

Group III (n = 14): interactional pattern of explanation. The third and largest group was characterized by high percentages of experiencing aggressiveness and tension, having struggles with fellow patients, getting along with the therapists and pursuing a two-fold strategy. Similar to the first group, negative influences by fellow patients are mentioned by every second patient, while only one patient reported solidarity with the drug milieu as a reason. Another parallel to group II is the absence of substance consumption, being mentioned only once.

**Table 2** shows the comparison of the three cluster groups on some non-construct-related variables. The test of the distribution of main diagnoses reveals no significance. However, descriptively, some peculiarities attract attention: all included patients with cocaine-associated disorder belong to group III, and alcohol-associated disorders are very prominent in group II. Combining primary and secondary polytoxic addiction diagnoses, all group I patients except for one are diagnosed as demonstrating polytoxic addiction patterns. Secondary personality disorders are not related to the cluster groups, while they are, at a descriptive level, slightly overrepresented in group III. The same holds true for the number of secondary somatic diagnoses. 

**Table 2:** Patients’ characteristics according to cluster group; cluster differences (non-construct-related).

Variable/*specification*		Cluster group^1^			Test statistics^2^	
	Total	Substance related	Motivational deficiency	Interactional		
	n = 37	n = 7	n = 10	n = 14	Chi²	p³
Diagnoses						
Main diagnosis					8.3	.22
*Polytoxic (F19)*	24%	43%	10%	36%		
*Cocaine-related (F14)*	11%	–	–	21%		
*Opioid-related (F11)*	32%	29%	30%	21%		
*Alcohol-related (F10)*	32%	29%	60%	21%		
Additional secondary diagnosis						
*Polytoxic (F19)*	24%	43%	20%	21%	1.4	.50
*Personality disorder*	16%	14%	10%	29%	1.4	.49
*No. of somatic diagnoses*	.8 ( ± 1.4)	.7 ( ± 1.1)	.9 ( ± 1.6)	1.1 ( ± 1.6)	.08	.96
Legal aspects						
Main offence					5.6	.85
*Killing (incl. attempted)*	3%	–	10%	–		
*Violent assault*	41%	57%	50%	43%		
*Sexual offence*	5%	–	10%	7%		
*Robbery/theft/fraud*	14%	–	10%	7%		
*Drug offence*	35%	43%	20%	36%		
*Other offence*	3%	–	–	7%		
No diminished liability	59%	71%	30%	57%	3.1	.21
Concurrent prison sentence (month)	47.2 ( ± 24.9)	45.4 ( ± 15.3)	32.7 ( ± 14.2)	56.4 ( ± 25.7)	6.3	.04*
Criminal history						
Previously served prison sentences (month)	37.9 ( ± 43.5)	19.9 ( ± 18.2)	25.1 ( ± 28.3)	55.1 ( ± 55.5)	5.9	.05°
Entries in police files	12.7 ( ± 7.4)	7.7 ( ± 2.5)	13.5 ( ± 6.8)	13.4 ( ± 5.9)	6.6	.04*
Age at first delinquency	20.0 ( ± 7.3)	19.7 ( ± 6.3)	21.6 ( ± 9.8)	18.9 ( ± 6.1)	1.0	.61
Sociodemography						
Marital status					15.1	.005**
*Single*	65%	57%	100%	43%		
*Married*	16%	43%	–	14%		
*Divorced*	19%	–	–	43%		
Age at admission	35.9 ( ± 9.6)	29.1 ( ± 6.5)	37.2 ( ± 11.2)	35.9 ( ± 9.9)	2.3	.31
Treatment duration (months)	14 ( ± 9.5)	21.8 ( ± 13.5)	12.6 ( ± 5.5)	13.8 ( ± 8.6)	2.3	.31

^1^n = 6 missing values due to exclusively 0-values or n > 3 single missing values.

^2^Kruskal-Wallis test/chi-square test for linear data; crosstab s for categorical data.

^3^° if p < .1, * if p < .05, ** if p < .01.

Concerning legal aspects, the type of offence is not related to the cluster group, but the percentage of patients without diminished liability differs on a descriptive level, and the duration of the concurrent prison sentence varies significantly among the groups. As this indicator serves as an estimator of offence severity, it is very probable that patients with interactional explanation patterns committed the most severe offences.

Groups differ on variables indicating criminal history as well. On a trend level, it is again group III that attracts attention. It shows the highest prior prison experience as a trend, high numbers of entries in police files (similar to group II on this measure) and the lowest age descriptively at first delinquency.

Very strong relationships can be seen with respect to marital status: every group II patient is single, while all divorced patients pertain to group III. Descriptively, group I patients were 7 years younger at admission than patients in groups II or III, while the average treatment duration of this group lasted 8 months longer than that of groups II and III.

## Discussion

The study intended to shed some light on the differences between therapists’ and patients’ perspectives concerning the treatment course leading to therapy failure in the particular context of German forensic addiction treatment according to sec. 64 *StGB*. The comparison of patients’ and therapists’ perspectives revealed numerous and strong differences, most likely indicating an incapacity to establish a common frame of reference for assessing therapy processes. This could be one of the major reasons why treatment dynamics take on a life on their own towards a disruption of the therapeutic relationship, leading to therapy failure. To avoid this outcome, patients and therapists should be encouraged to monitor their thoughts and feelings in relation to treatment on a regular basis beginning early in the therapy process. Clear differences should be viewed as evidence that more work is needed to improve therapeutic relationships. However, much more research focusing on the meaning of differences in patients’ and therapists’ perspectives is needed to exclude potential biases deriving from emotional overlay or motivational factors.

The control group served a small group of patients with regular therapy termination. Interestingly, the comparison of this group to patients with therapy failure showed that different ratings on the possible causes and occasions for premature therapy termination were rare—except for statements denoting the therapeutic relationship or working alliance. These factors were assessed much better by successful patients. The findings go along with evidence from general psychotherapy research (24, 25) and underline the importance of establishing a supporting and trustful therapeutic relationship as a precondition for successful forensic addiction treatment. They therefore probably pose the major challenge for forensic psychotherapy.

Negative emotions were frequently expressed in patients’ narratives explaining their subjective concept of therapy failure. The vast majority described feelings of disappointment or used even stronger terms to express their anger and rage. As the interviewed patients all awaited their referral to prison, this observation calls for a supportive and stabilizing therapeutic approach at the end of treatment rather than the continuation of a confronting therapeutic style that surely would not “heal” the broken relationship but rather would risk the intensification of negative attitudes towards professional help and support.

The second intention of the present analysis was the replication of the cluster results derived from our pilot study (17). The present cluster structure did fit ambivalently to the cluster structure revealed there (which was not based on patients’ ratings but rather on an analysis of clinical files): the pattern of passive refusal corresponds quite well to the motivational deficiency pattern, while the patterns of confronting acting out and of impulsive refusal can only be marginally related to the motivational deficiency pattern and the interactional pattern of explanation. It can be assumed that, in addition to clear passive and withdrawing behaviors, patients develop patterns of explanations that differ from those of the hospital, or they weigh the explanatory patterns differently.

However, the revealed cluster structure makes sense from a therapeutic viewpoint, as it groups phenomena in a way that fits clinical experience: notwithstanding that even “coerced” addiction therapy is effective (26), every addiction therapist knows patients who cannot profit even from long-lasting therapies, as their addiction is simply too ingrained—the “prototype” of a patient of the substance-related group. The type of patient with moderate criminal and addictive behavior but major motivational problems (as can be found in the motivational deficiency group) also appears familiar in the forensic context. The same holds true for patients challenging the ward with interactive peculiarities—in other words, typical “troublemakers” [(27) as an early and critical description of this phenomenon].

Nevertheless, it would be short-sighted to restrict the discussion to the patients’ personal characteristics. The described prototypical behaviors and problems can be seen as sets of possible risk factors impairing the therapeutic course, which is surely a product of both parties’ behaviors. The earlier the risk factors are identified, the better, because they allow specific therapeutic attitudes and strategies to take appropriate countermeasures, i.e., to impede the critical dynamics at the beginning.

It is no surprise that patients restage their life problems—whether they are substance consumption or relational problems—during therapy in a way such that they also dominate the therapeutic course. However, it is striking that the results of cluster analysis underline this assumption so strongly, as (despite the small sample size) the typical treatment dynamics towards therapy failure are connected to several characteristics beyond the actual treatment situation: diagnostic and legal factors, as well as criminal history or sociodemographic information, differ between the cluster groups. In accordance with the previously outlined risk model, therapists can use this information (accessible upon patient admission) to specifically prepare the treatment course or to at least become aware of possible disturbances.

### Limitations

The present paper is based on a retrospective study with a relatively small sample size and did not use standardized materials, which surely limits the reliability of our findings. The unique German legal concept of a custodial addiction treatment order restricts the degree to which these results can be generalized.

However, the complex methodology (combination of within-group and between-group design; the application of both semistructured interviews and questionnaires) and very conservative nonparametric test statistics allow for prudent and explorative insights into the treatment dynamics of German forensic addiction treatment, which have not yet been scientifically addressed.

Further and more elaborate research is certainly needed, as our study is only the first small step towards a real understanding of therapy dynamics within forensic addiction treatment.

## Data Availability Statement

The datasets generated for this study are available on request to the corresponding author.

## Ethics Statement

This study was carried out in accordance with the Declaration of Helsinki. As it was primarily an internal evaluation of quality management, no ethical committee was involved. All subjects were informed about the secondary aim to use their data for research purposes and gave written informed consent.

## Author Contributions

JQ and KH contributed to the conception and design of the study. JQ was the researcher responsible for the field work, the organization of the database and the performance of the data analyses. JQ and LL wrote the first draft of the manuscript. All authors contributed to the revision and read and approved the submitted version.

## Funding

The research was funded by the German Federal State of Baden-Württemberg.

## Conflict of Interest

The authors declare that the research was conducted in the absence of any commercial or financial relationships that could be construed as a potential conflict of interest.
